# From Molecules to Medicine: Deciphering Obesity and Lipid Metabolism for Translational Insights

**DOI:** 10.3390/biomedicines14010068

**Published:** 2025-12-29

**Authors:** Sandeep Kumar, Abhishek Gupta

**Affiliations:** 1 Independent Researcher, New Orleans, LA 70112, USA; 2 Department of Biochemistry and Structural Biology, Joe R. and Teresa Lozano School of Medicine, University of Texas at San Antonio, San Antonio, TX 78229, USA

## Abstract

Obesity, type 2 diabetes (T2D), and insulin resistance are pervasive metabolic disorders marked by chronic low-grade inflammation and systemic metabolic disorders. The emerging field of immunometabolism highlights how interactions between immune processes and metabolic pathways in adipose tissue, liver, muscle, and pancreatic islets contribute to disease pathogenesis. Lipid dysregulation plays a central role in these processes, with distinct lipid molecules identified in obese patients as compared to lean patients that correlate with insulin resistance, inflammation, and vascular dysfunction. This Special Issue compiles a multidisciplinary body of research aimed at elucidating molecular mechanisms, identifying novel biomarkers, and exploring innovative therapeutic strategies. Key contributions include studies on omega-3 long-chain polyunsaturated fatty acids (LCPUFAs) and their differential associations with neurocognitive development; the potential of beta-defensin 2 as a biomarker linking gut-derived inflammation and metabolic dysfunction; and the promotion of adipocyte browning by Carnosic acid via AMPK activation and GSK3β inhibition. Additionally, reviews of phytochemicals underscore their multisystem therapeutic potential, while investigations into sodium–glucose cotransporter-2 (SGLT2) inhibitors suggest possible metabolic and neuroprotective benefits beyond glucose control. Maternal lipid metabolism during pregnancy and its impact on maternal fetal health further emphasize the clinical complexity of lipid dysregulation. Despite promising insights, significant gaps remain regarding causality versus correlation in lipid biomarkers, standardization of analytical methodologies, tissue heterogeneity, and unintended effects of metabolic interventions. Collectively, these studies underscore the necessity of integrative, mechanism-driven research to bridge fundamental biology with translational and clinical applications, ultimately advancing precision therapies for metabolic diseases.

## 1. Introduction

Chronic tissue inflammation has emerged as a key feature of obesity and T2D and is observed in insulin target tissues, such as adipose tissue, liver, muscle, and pancreatic islets [1,2]. These observations have led to the term “immunometabolism”, which incorporates the underlying interplay between immunologic processes and metabolic defects [2,3]. Metabolic disorders, obesity, type 2 diabetes, and insulin resistance are multifactorial and dynamic. Addressing these issues successfully demands multifaceted, integrated strategies, and critical challenges for global health [4,5], whether through molecular engineering, precision drug delivery, multifunctional compounds, or integrative strategies combining lifestyle, biologics, or devices. Obesity, type 2 diabetes, and insulin resistance remain major public health challenges, with the burden growing worldwide [6,7,8]. The complex interplay of genetics, environment, cell signaling, and metabolic homeostasis underlies obesity, type 2 diabetes, and insulin resistance [9,10].

Deciphering the molecular underpinnings of lipid dysregulation holds great promise not only for deepening mechanistic understanding but for enabling more precise, effective, and durable therapies for obesity, insulin resistance, type 2 diabetes, non-alcoholic fatty liver disease (NAFLD/MASLD), and related complications [11,12]. Profiling the lipid content of adipose tissue, secreted vesicles (adiposomes), and circulating lipids has revealed distinct “lipid signatures” in obese vs. lean states. These signatures correlate with insulin resistance, systemic inflammation, and vascular dysfunction, suggesting that some lipid species may serve both as biomarkers and as mediators of disease [13,14,15]. Lipid metabolism in adipose tissue, liver, skeletal muscle, the gut, and perhaps even the nervous system is tightly linked via molecules like adipokines [16], free fatty acids, ceramides, and via signaling pathways such as TGF-β and inflammatory/NLRP3-driven inflammation [17,18]. The present research topic seeks to confront them with creativity, precision, and innovation. We are particularly interested in research that identifies novel molecular players, unravels previously unrecognized pathways, and pioneers strategies for more selective, potent, and efficient therapies.

## 2. Summary of the Special Issue

Obesity and lipid dysregulation remain central challenges in global health, contributing to a cascade of metabolic, inflammatory, cardiovascular, neurocognitive, and reproductive complications. This Special Issue brings together a diverse collection of studies that span molecular mechanisms, biomarker discovery, nutraceutical interventions, and clinical implications illustrating the multidimensional landscape of obesity research. Collectively, these works bridge fundamental biology with translational potential, uncovering how lipids, inflammation, and metabolic signaling shape human health from childhood development to pregnancy and aging.

### 2.1. Omega-3 Lipids and Neurocognitive Processing

This study indicates that specific omega-3 LCPUFAs (Long-Chain Polyunsaturated Fatty Acids) may play different roles in children’s reading and writing development. EPA (eicosapentaenoic acid, 20:5 n-3) was linked mainly to skills involving sound processing and visual perception, while DHA (docosahexaenoic acid, 22:6 n-3) showed stronger ties to visual attention. These cognitive abilities were related to reading speed, reading accuracy, and writing accuracy, but only the auditory phonological domain clearly explained how omega-3 LCPUFA levels connect to better literacy performance. EPA stood out for its role in reading speed, and both EPA and DHA were associated with fewer reading and writing errors. At the same time, the findings reflect the broader literature showing that omega-3 LCPUFAs can have mixed effects depending on factors like dose, age, and individual differences.

The study also revealed that the interaction between fatty acids matters. Higher DHA levels reduced the negative direct influence of EPA on reading time, allowing EPA’s indirect benefits through stronger auditory processing to support faster reading. The balance of AA and ALA further shaped how EPA affected reading speed, with higher ALA linked to more favorable outcomes. Differences in how reading speed and accuracy responded suggest that omega-3s may support both whole-word recognition and sound-based decoding. Overall, the study suggested that omega-3 LCPUFAs may play an important role in the brain processes that help children learn to read and write, with EPA having the strongest influence and other fatty acids helping shape that impact.

### 2.2. Gut-Derived Inflammation and Novel Biomarkers in Obesity

Recently, gut microbiome has been shown to influence many physiological/metabolic processes, fat accumulation/obesity being one of them. Obesity is often accompanied with chronic low-grade inflammation. Beta-2-defensin is a peptide involved in immune defense and has a role to play in gut microbial balance. However, the contribution of beta-defensin 2 has not been well characterized in obesity. Studies reaffirm that obesity is associated with increased systemic inflammation, reflected in elevated hs-CRP levels, and that beta-defensin 2 was positively correlated with presepsin in overweight individuals. This suggested a connection between gut microbial imbalance and the inflammatory state in obesity, highlighting the potential of beta-defensin 2 as a biomarker linking these processes.

Overall, these observations provided initial evidence that beta-defensin 2 might be involved in the immune and inflammatory changes associated with obesity. By offering insight into the interplay between gut microbiota and systemic inflammation, these findings contributed to a better understanding of the mechanisms driving metabolic dysfunction and suggested directions for future research and targeted therapeutic strategies.

### 2.3. Browning in Adipocytes: A Potential Strategy for Obesity Management

Amid the growing burden of obesity and metabolic disorder in the current population, identification of the compounds that can transform energy-storing white fat into energy-burning beige fat has emerged as a promising strategy. In search of new approaches to tackle obesity and related metabolic disorders, a group of researchers investigated whether Carnosic acid (CA) could encourage white fat cells to adopt the characteristics of energy-burning beige cells. In experiments with 3T3-L1 adipocytes, CA treatment led to striking changes: levels of key browning markers, including UCP-1, PRDM16, and PPARγ, increased significantly, while proteins involved in mitochondrial growth, such as PGC-1α and TFAM, were also elevated. The cells had increased mitochondrial content and reduced lipid accumulation, indicating a shift toward enhanced energy expenditure. Interestingly, many of these effects were like those produced by metformin, a drug known to promote browning and enhances mitochondrial function in fat cells.

Further exploration revealed the mechanisms behind these effects. CA activated AMPK and its downstream target ACC while inhibiting GSK3β, a known suppressor of browning. The blockade of AMPK diminished the browning and lipid-reducing effects of CA, highlighting AMPK’s central role in mediating these changes. These findings suggest that the combined action of AMPK activation and GSK3β inhibition drives the conversion of white fat to a beige, energy-expending state. Overall, the authors show that Carnosic acid promotes the browning of adipocytes and reduces lipid accumulation through AMPK activation and GSK3β inhibition, highlighting its potential as a therapeutic strategy against obesity.

### 2.4. Phytochemicals as Multisystem Therapeutics

Chronic conditions such as diabetes, cancer, cardiovascular diseases, and infectious diseases exert a substantial global health burden. For decades, medicinal plants have been used in the traditional medical systems, including Ayurveda, Unani, Traditional Chinese Medicine, and European ethnomedicine. In one of the review articles, the authors highlight 35 medicinal plants with potential to manage major metabolic and inflammatory diseases. Studies demonstrated that these plants are rich sources of bioactive compounds with diverse therapeutic properties, encompassing antidiabetic, anticancer, antimicrobial, cardioprotective, anti-inflammatory, and immunomodulatory effects. For example, cucurbitane triterpenoids, diosgenin, and limonoids have been linked to improved glucose metabolism, while capsaicin and curcumin modulate cancer-related signaling pathways. Piperine, berberine, and allitridin exhibit antimicrobial activity, and flavonoids such as quercetin and genistein provide anti-inflammatory benefits. Ginsenosides show cardioprotective potential, whereas quercitrin and tannins support gastrointestinal function, acting via mechanisms including GLUT4 translocation, NF-κB inhibition, cell cycle arrest, PPARα and PI3K/Akt modulation, and COX/lipoxygenase suppression.

Despite great experimental success, the integration of plant-based therapies into conventional clinical practice still faces multiple challenges. The challenges could be attributed to their low bioavailability, delayed action, and variability in potency. Emerging strategies such as nanotechnology, biomarker profiling, multi-omics approaches, and artificial intelligence offer new ways to enhance targeted delivery and elucidate molecular mechanisms. By combining traditional knowledge with modern scientific tools, medicinal plants could prove to be a new, more potent mode of treatment. Integrating traditional medicinal plants with modern scientific approaches holds promise for developing precise, effective, and personalized therapies for chronic diseases.

### 2.5. Lipid Metabolism, Diabetes, and Neurodegeneration

SGLT2 inhibitors were originally developed to manage type 2 diabetes and later approved for select patients with type 1 diabetes, but their therapeutic scope has expanded considerably with strong evidence supporting cardiovascular and kidney protection. Beyond reducing blood glucose, they have been shown to lower the risk of heart failure and chronic kidney disease, and emerging research hints at broader effects on vascular health, coagulation pathways, and bone metabolism. Their potential influence on cognitive decline has also drawn attention, as multiple observational studies and pooled analyses have shown lower rates of dementia among users compared with non-users. Still, the overall picture remains inconclusive, since several clinical studies have not demonstrated significant cognitive improvements, likely due to short observation periods or inclusion of individuals with advanced cognitive impairment. Comparisons with other glucose-lowering medications produce variable results, with some data favoring GLP-1 receptor agonists or metformin in terms of cognitive benefit.

This review highlights a range of experimental findings pointing to biological mechanisms through which SGLT2 inhibitors could support brain health. Proposed pathways include improved microvascular circulation in the brain, attenuation of oxidative stress and inflammatory responses, enhanced mitochondrial activity, and support for neuronal repair and structural remodeling. These mechanisms may help counter hallmarks of dementia such as amyloid buildup, tau abnormalities, and glucose-driven neuronal injury. Although these insights are promising, clinical evidence remains limited and largely observational, and no randomized controlled trials have yet validated these neuroprotective effects. Overall, the review suggests that SGLT2 inhibitors could play a role in reducing or slowing cognitive impairment in individuals with diabetes, but well-designed prospective studies would be needed particularly to determine whether similar benefits extend to people with normal glucose regulation.

### 2.6. Maternal Lipid Metabolism and Pregnancy Outcomes

Pregnancy naturally causes lipid levels to rise, but identifying when these changes become excessive is important for protecting both mother and baby. While most increases are normal, very high lipid levels can signal underlying problems and raise the risk of complications such as pancreatitis, severe nausea, or metabolic issues. Women with a history of lipid disorders, symptoms of metabolic imbalance, or conditions like gestational diabetes should be monitored more closely. Case reports show that treatment approaches vary from diet changes to lipid-lowering medications and, in severe cases, insulin therapy but clear, standardized guidelines are still lacking. Larger studies are needed to determine when to screen, how to treat safely, and what long-term effects to expect.

Genetics, insulin resistance, and the added metabolic demands of pregnancy can worsen lipid abnormalities, increasing risks for both the mother and fetus. Although rare, severe hypertriglyceridemia can lead to acute pancreatitis, underscoring the need for early recognition and prompt care. Proper evaluation through symptoms, blood tests, and targeted diagnostics helps distinguish normal pregnancy-related changes from harmful conditions. Coordinated care among obstetricians, endocrinologists, and gastrointestinal specialists is often necessary for effective management. Overall, early detection, personalized treatment, and a team-based approach are key to reducing complications. This review emphasizes the need to clearly separate normal from harmful lipid changes in pregnancy and highlights the importance of further research to guide safe and effective care.

## 3. Gap in Knowledge

Several major concerns highlighted throughout this Special Issue underscore the complexity of metabolic disorders such as obesity, diabetes, and cardiovascular complications, and the need for deeper mechanistic understanding. First, although numerous lipidomic biomarkers exhibit strong correlations with obesity, insulin resistance, and type 2 diabetes, the field still lacks clarity on whether these lipid species play causal roles in disease progression or simply serve as downstream markers of metabolic dysfunction. Disentangling causality from correlation remains a critical challenge.

Second, there is insufficient standardization across analytical platforms and measurement methodologies, which hampers reproducibility and cross-study comparisons. Moreover, the validation of emerging lipid and regulatory biomarker candidates is limited by the scarcity of large, well characterized cohorts that represent diverse ethnicities, ages, and sexes an essential requirement for establishing robust, generalizable biomarkers.

Third, the inherent heterogeneity among tissues and cell types poses another major obstacle. Differences between visceral and subcutaneous fat depots, distinct adipocyte subpopulations, and the presence of various immune and stromal cell types contribute to depot-specific metabolic responses. This cellular diversity complicates the interpretation of biomarker data and the development of targeted interventions.

Finally, interventions aimed at modulating lipid metabolism, whether dietary, pharmacological, or lifestyle-based, often exert broad and sometimes unintended effects. Our current understanding of off-target or compensatory responses remains incomplete, making it challenging to predict therapeutic outcomes and design highly specific treatments.

Together, these issues highlight the pressing need for standardized methodologies, comprehensive multi-centric cohorts, improved mechanistic studies, and more precise therapeutic strategies to advance the field of metabolic disease research.

## 4. Future Perspectives and Conclusions

This Special Issue reflects the ongoing evolution of obesity research, moving beyond the identification of molecular signatures toward the development of targeted interventions that harness metabolic pathways for personalized medicine. Collectively, the studies presented here underscore that advancing obesity and lipid metabolism research requires a comprehensive approach, one that integrates mechanistic insights with translational and clinical strategies. Such integration is essential for bridging the gap between bench discoveries and meaningful improvements in patient care, ultimately paving the way for more precise, effective, and individualized therapeutic options for metabolic diseases.
